# An ion-channel-gene-based prediction model for head and neck squamous cell carcinoma: Prognostic assessment and treatment guidance

**DOI:** 10.3389/fimmu.2022.961695

**Published:** 2022-10-28

**Authors:** Yanxun Han, Yangyang Shi, Bangjie Chen, Jianpeng Wang, Yuchen Liu, Shuyan Sheng, Ziyue Fu, Chuanlu Shen, Xinyi Wang, Siyue Yin, Haiwen Li

**Affiliations:** ^1^ Department of Otolaryngology, Head and Neck Surgery, The First Affiliated Hospital of Anhui Medical University, Hefei, Anhui, China; ^2^ Anhui Medical University, Hefei, Anhui, China; ^3^ Department of Emergency Surgery, The First Affiliated Hospital of Anhui Medical University, Hefei, Anhui, China; ^4^ Department of Oncology, The First Affiliated Hospital of Anhui Medical University, Hefei, Anhui, China; ^5^ Department of Gastroenterology, The First Affiliated Hospital of Zhengzhou University, Zhengzhou, China

**Keywords:** head and neck squamous cell carcinoma, ion channel, prognosis, immune infiltration, chemotherapy sensitivity

## Abstract

**Purpose:**

Head and neck squamous cell carcinoma (HNSCC) is a very diverse malignancy with a poor prognosis. The purpose of this study was to develop a new signature based on 12 ion channel genes to predict the outcome and immune status of HNSCC patients.

**Methods:**

Clinicopathological information and gene sequencing data of HNSCC patients were generated from the Cancer Genome Atlas and Gene Expression Omnibus databases. A set of 323 ion channel genes was obtained from the HUGO Gene Nomenclature Committee database and literature review. Using univariate Cox regression analysis, the ion channel genes related to HNSCC prognosis were identified. A prognostic signature and nomogram were then created using machine learning methods. Kaplan-Meier analysis was used to explore the relevance of the risk scores and overall survival (OS). We also investigated the association between risk scores, tumor immune infiltration, and gene mutational status. Finally, we detected the expression levels of the signature genes by quantitative real-time polymerase chain reaction, western blotting, and immunohistochemistry.

**Results:**

We separated the patients into high- and low-risk groups according to the risk scores computed based on these 12 ion channel genes, and the OS of the low-risk group was significantly longer (p<0.001). The area under the curve for predicting 3-year survival was 0.729. Univariate and multivariate analyses showed that the 12-ion-channel-gene risk model was an independent prognostic factor. We also developed a nomogram model based on risk scores and clinicopathological variables to forecast outcomes. Furthermore, immune cell infiltration, gene mutation status, immunotherapy response, and chemotherapeutic treatment sensitivity were all linked to risk scores. Moreover, high expression levels of ANO1, AQP9, and BEST2 were detected in HNSCC tissues, whereas AQP5, SCNN1G, and SCN4A expression was low in HNSCC tissues, as determined by experiments.

**Conclusion:**

The 12-ion-channel-gene prognostic signatures have been demonstrated to be highly efficient in predicting the prognosis, immune microenvironment, gene mutation status, immunotherapy response, and chemotherapeutic sensitivity of HNSCC patients.

## Introduction

Head and neck cancer involves a very aggressive group of tumors, with squamous cell carcinoma accounting for 90% of patients (1). Head and neck squamous cell carcinoma (HNSCC) is the 6th most common cancer worldwide, with 890,000 new cases and 450,000 deaths in 2018 (2, 3). The prevalent risk factors for HNSCC include genetic factors, smoking, alcohol intake, and viral infections (4, 5). Most patients with HNSCC are locally advanced, primarily treated with surgery, chemotherapy, radiotherapy, and molecularly targeted therapy as needed (6, 7). Despite advancements in current therapeutic options, the prognosis of HNSCC patients has remained unchanged (7). Due to the diversity of anatomical locations and the complexity of etiology, the biological characteristics of HNSCC vary widely, and the sensitivity to treatment also varies from person to person. Therefore, it is crucial to logically stratify HNSCC patients so that specific treatment regimens can be instituted to improve their prognosis.

Ion channels are transmembrane proteins that selectively transport ions across cell membranes. Ion channels are present in nearly every cell and function in various physiological activities, including excitability, cell cycle progression, contraction, and metabolism. Therefore, defects in ion channel function impair key cellular processes, resulting in various diseases (8). Unsurprisingly, the incidence of diseases associated with ion channel defects (also known as "channel diseases") has skyrocketed in recent years (8–10). Ion channels are also relevant to tumor cell biology; for example, they play an important role in regulating gene expression, cell migration, cell proliferation, and other tumor pathophysiological processes (11, 12). Research has been conducted to better understand the potential mechanisms of ion channels in tumors, among which ion channels have been found to be associated with tumor immunomodulation. Under normal conditions, ion channels are abundantly expressed in immune cells and play an essential role in maintaining immune cell activity, regulating lymphocyte development, and modulating immune response (13, 14). For example, in the case of Ca+ channels, the activation and effects of immune cells depend on Ca+ influx. By regulating changes in intracellular calcium ion concentrations, T cell maturation and anti-tumor cytotoxic T lymphocyte responses can be promoted (15). In the tumor immune microenvironment, ion channel dysfunction and inactivation of calcium ion channels that recognize tumor antigens affect tumor cell anti-cancer immunity, and tumor cell hyperpolarization increases the possibility of immune evasion and promotes tumor progression. In summary, the value of ion channels in tumor diagnosis, prognosis, and as targets for membrane therapy has received widespread attention (10, 16, 17).

In addition, immune checkpoint inhibition (ICI) has been introduced into the treatment regimen for metastatic HNSCC, suggesting that ICI is a viable treatment option for HNSCC. However, some patients develop drug resistance and disease progression after receiving ICI therapy, which may involve tumor-infiltrating immune cells (18). Previous studies have indicated that ion channels can promote HNSCC progression by modulating the tumor microenvironment (19). They found that a decrease in KCNA3, which encodes potassium voltage-gated channels in tumor-infiltrating lymphocytes, leads to a decrease in the immunosurveillance of HNSCC (19). Enhancing the activity of Kv1.3 and KCa3.1, channels related to immunosuppression, may improve the immune response of HNSCC patients (12, 19). Therefore, further understanding the relationship between the tumor microenvironment and ion channels will help improve the outcomes of HNSCC patients treated with ICI.

We developed a 12-ion-channel-gene signature using integrative analysis to predict the prognosis of patients with HNSCC. In The Cancer Genome Atlas (TCGA) dataset, Least Absolute Shrinkage and Selection Operator (LASSO)-Cox analysis determined 12 genes with non-zero regression coefficients (ANO1, AQP1, AQP5, AQP9, BEST2, CHRNA5, KCNJ15, KCNG1, SCNN1G, SCN4A, TRPC1, and VDAC1). Based on these genes, we established the signature, which was verified using the Gene Expression Omnibus (GEO) dataset. Furthermore, we created a simple nomogram based on the risk scores and other clinical features. In addition, we explored the associations between risk scores and tumor mutation frequency, immune infiltration, immunotherapy sensitivity, and chemosensitivity.

## Methods

### Data collection

The TCGA GDC portal (https://portal.gdc.cancer.gov) was used to download gene expression and clinical data for HNSCC (20). GEO (http://www.ncbi.nlm.nih.gov/geo/) provided the GSE65858 (21) and GSE41613 (22) chips. The "sva" tool was used to remove batch effects that occur when combining large datasets (23). The expression values of the datasets (TCGA, GSE65858, and GSE41613) were transformed using log2 before normalization across platforms. The comBat algorithm was then used to reduce the batch effects between the three datasets. We next screened and recorded 360 ion channel-related genes through an extensive literature review (24–26) and then intersected 330 genes downloaded from the HUGO Gene Nomenclature Committee (HGNC) database (https://www.genenames.org/data/genegroup/#!/group/177), resulting in a total of 323 distinct ion channel-related genes. We analyzed these genes using univariate Cox regression to identify genes with prognostic significance for future studies. All data used in this study were freely accessible.

### Cluster analysis of ion channel genes

We first explored the function and prognostic significance of ion channel genes in patients with HNSCC. After cluster analysis using the K-means algorithm to determine an appropriate number of clusters, we utilized consensus non-negative matrix factorization (CNMF) techniques to classify HNSCC samples into different molecular cancer subtypes. We used the ESTIMATE, CIBERSORT, and MCPcounter algorithms to determine immune scores and immune cell expression levels for different subtypes and investigated the differences in immune infiltration status among subtypes. The functions of differentially expressed genes (DEGs) among subtypes were investigated using the Kyoto Encyclopedia of Genes and Genomes (KEGG) and Gene Ontology (GO) enrichment analysis.

### Construction and evaluation of ion channel genes signature for HNSCC

LASSO analysis was used in the TCGA cohort to further filter out genes associated with prognosis according to the results of the univariate Cox analysis. The ion channel gene risk signature was then developed using the following formula: score = (gene 1 expression * correspondence coefficient) + ...+ (gene n expression * correspondence coefficient). Subsequently, we determined all patients’ risk scores using the formula above and divided the patients into high- and low-risk groups based on the median risk score. The "survival" and "survminer" R packages were utilized to analyze and compare overall survival (OS) differences between two groups in the models, and the prognostic power of the model was estimated using time-dependent receiver operating characteristic curves (ROCs). The same procedure was performed for the GSE65858 and GSE41613 datasets to test the reliability of the signature. Then, we compared the model's predictions with those of Jiang (27), Liu (28), and Zhang (29). In addition, we also compared the prognostic value between the risk signature and traditional models (age, gender, T, N, stage, and grade). Finally, signature independence was assessed using univariate and multivariate Cox regression analyses.

### Development and assessment of nomogram

We then created a nomogram model combining risk scores and clinicopathological parameters to predict the prognosis of patients with HNSCC. To assess the precision of the nomogram, a calibration curve and concordance index (C-index) were drawn and analyzed separately. The clinical value of the nomogram was further estimated using decision curve analysis (DCA).

### Mutation analysis of ion channel genes associated with prognosis

The 'RCircos' program in R was used to locate loci of copy number variation (CNV) changes in prognosis-relevant ion channel genes on 23 chromosomes. Additionally, the somatic mutation information in the TCGA database was analyzed using the "maftools" package to compare the mutation landscape differences between high- and low-risk groups.

### Comprehensive correlation analysis of immune infiltration

To investigate the link between risk scores and the immune microenvironment, we considered widely recognized techniques for assessing immune cell infiltration, including XCELL, TIMER, QUANTISEQ, MCPcounter, EPIC, CIBERSORT-ABS, and CIBERSORT. Differences in immune-infiltrating cell content between the two groups were examined using the Wilcoxon test; the findings are shown in boxplots and heat maps. The relationship between risk scores and immune infiltration was investigated using Spearman analysis. The lollipop plot depicts the correlation coefficients of the results. In addition, we used the ESTIMATE algorithm to evaluate the difference in the immune infiltration status between the high- and low-risk groups.

### KEGG and GO analysis

Similar to the above, we used the “Limma” package to explore the DEGs in the low- and high-risk groups; the thresholds were set as log FC >1, along with a false discovery rate <0.05. Then, we performed KEGG and GO analysis using the “clusterProfiler” R package, and the results were visualized with the “GOplot” package.

### Immunotherapy response prediction

We first estimated the tumor immune dysfunction and exclusion (TIDE; http://tide.dfci.harvard.edu) for each HNSCC patient in the TCGA dataset. TIDE is a computational framework that analyzes gene expression patterns of cancer patients to evaluate their response to immunotherapy and predict tumor immune escape (30). The lower the TIDE score, the better the response to immunotherapy. We compared the difference in TIDE scores between the high- and low-risk groups and the correlation between the risk and TIDE scores.

Additionally, we downloaded the Imvigor210 cohort (31), an immunotherapy cohort, to further verify the value of the ion channel gene signature in predicting immunotherapy response. The cohort included muscle-invasive bladder cancer tissues from patients treated with the anti-PD-L1 antibody atezolizumab (n=348). Differences in the responses to immunotherapy between the two groups were compared.

### Association analysis of risk scores and chemotherapy sensitivity

The half inhibitory concentration (IC50) represents the drug levels required for 50% inhibition of tumor cells. The lower the IC50 value, the more sensitive the drug is. The package “pRRophetic” can estimate the IC50 of chemotherapeutic drugs by constructing a ridge regression model based on public cell line expression profiles (32). In the TCGA cohort, the IC50 of 12 chemotherapeutic agents used to treat HNSCC patients, including bicalutamide, gemcitabine, sorafenib, docetaxel, doxorubicin, epothilone, parthenolide, shikonin, lenalidomide, metformin, vinorelbine, and methotrexate, was assessed using "pRRophetic" package. The difference in IC50 values between the two groups was investigated using the Wilcoxon test. The "ggpubr" and "ggplot2" packages were performed to generate boxplots.

### Tissue specimens

Fresh HNSCC and adjacent normal tissues were obtained from the First Affiliated Hospital of Anhui Medical University. This study was approved by the Ethics Committee of the First Affiliated Hospital of the Anhui Medical University (number: Quick-PJ 2022-03-19). None of the patients received anti-cancer treatment before surgery and signed an informed consent form. HNSCC and paracancerous tissues were obtained from each patient by an experienced pathologist.

### Real-time polymerase chain reaction assay

RNA levels were measured using real-time PCR. Briefly, Trizol was used to extract the mRNA, and Evo M-MLV RT Premix for quantitative PCR (qPCR) was used for reverse transcription. Quantitative real-time PCR was performed on an ABI 7500 real-time PCR thermocycler. Real-time PCR was performed using an SYBR®Green Premix Pro Taq HS qPCR Kit. Duplicate samples were used to compute the relative amounts of target transcripts after the data were normalized against GAPDH. Transcriptome levels were collected and differences were compared using the Wilcoxon test. The primer pairs used are listed in **Table S1**.

### Western blotting

Whole protein from the tissues was extracted using a RIPA lysis buffer. Protein content was determined using a BCA protein assay kit. A polyacrylamide gel with an SDS content of 10% was loaded with the protein extracts (20 g) and then transferred to a PVDF membrane. For two hours, these membranes were submerged in 5% (w/v) nonfat milk. Subsequently, the membranes were incubated with specific primary antibodies at 4 °C overnight. The following day, the membrane for an hour at room temperature with the appropriate secondary antibody. The membranes were washed again and examined using the ECL western blotting detection method.

### Immunohistochemistry

HNSCC and adjacent normal tissues were embedded in formalin and embedded in paraffin. Xylene was used to deparaffinize the paraffin-embedded human tissue sections, and graded ethanol was used to rehydrate the sections. Endogenous peroxidase activity was inhibited by treatment with 0.3% H_2_O_2_. The cells were primed with primary antibodies and treated overnight at 4 °C after blocking with a 5% BSA solution. After that, it was incubated for 30 min in a 37 °C water bath with goat anti-rabbit IgG labeled with horseradish peroxidase. The PBS was washed with PBS. An Olympus CX41 fluorescent microscope was used to visualize staining. Image J software was used to evaluate the results.

### Statistical analysis

All statistical analyses were performed using R (version 4.1.2) and GraphPad Prism (version 9.0.0). LASSO and Cox regression analyses were used to identify prognostic genes. Kaplan-Meier (K-M) analysis and log-rank tests were used to conduct survival studies. Associations were investigated using Spearman’s correlation analysis. Wilcoxon test was used to compare the two groups.

## Results

### Data preprocessing

**Figure 1:**
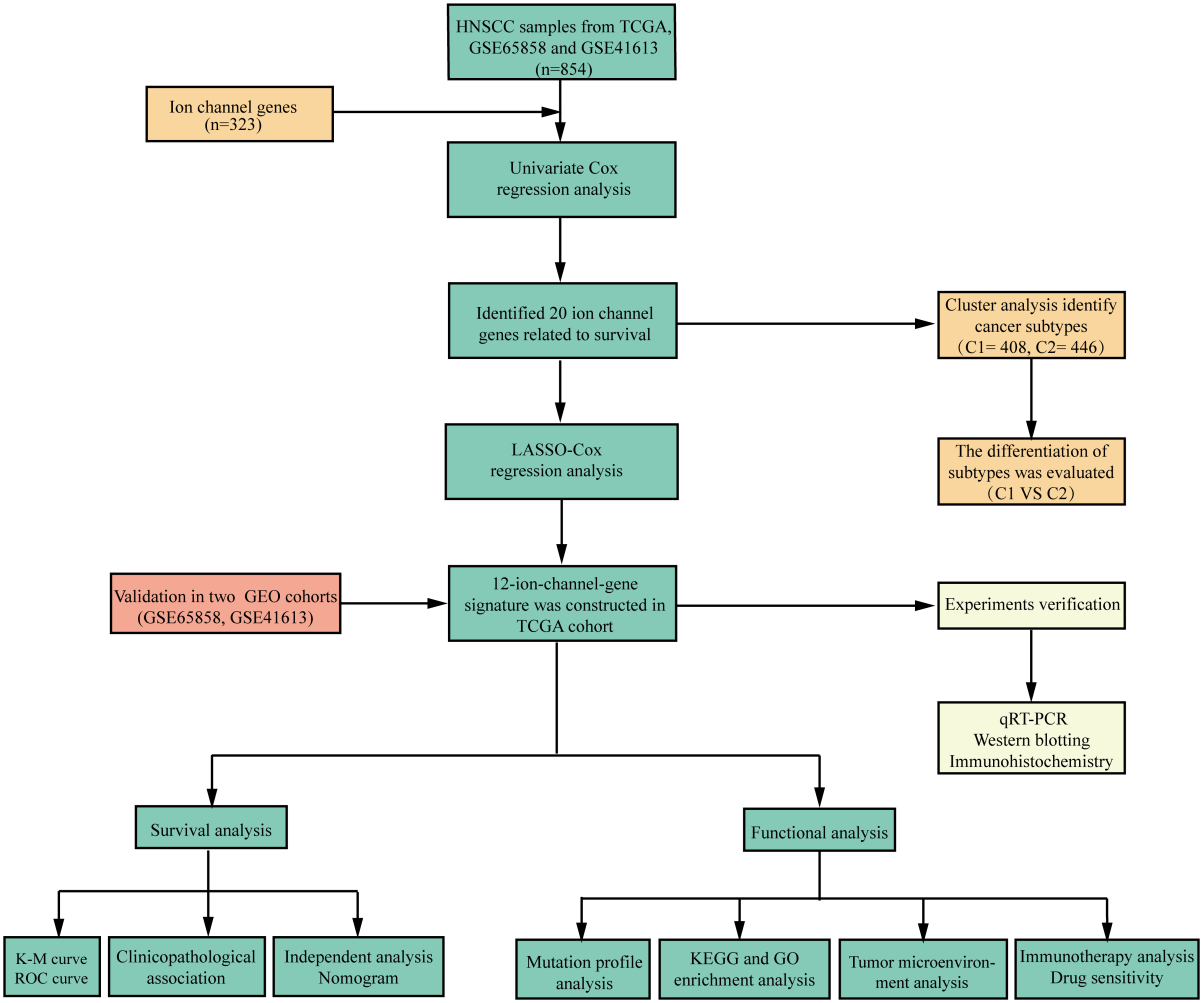
Flowchart of the study.

**Figure 2:**
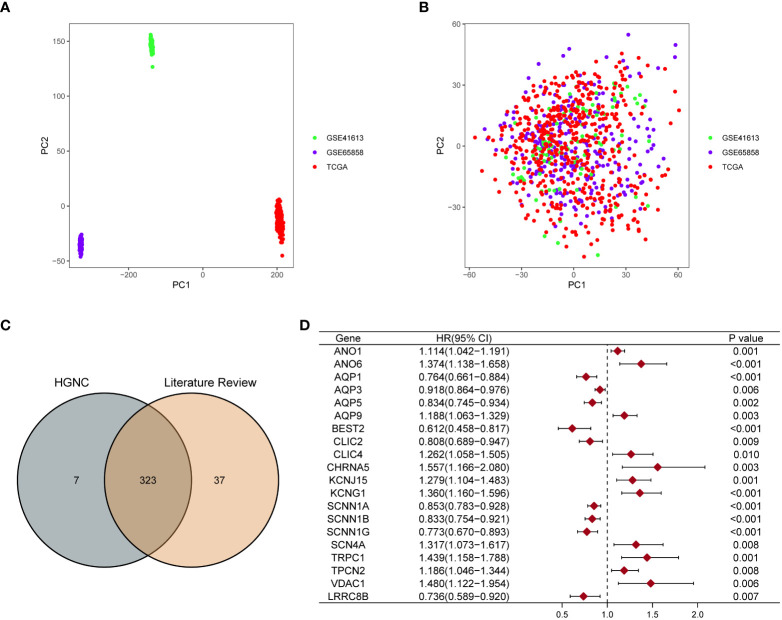
Screening ion channel genes related to the prognosis of HNSCC. PCA showed the distribution of three datasets before **(A)** and after removing the batch effect **(B)**. **(C)** Venn diagram showed that 323 ion channel genes were obtained by crossing the two gene sets. **(D)** The forest map showed that 20 ion channel genes related to the prognosis of HNSCC were identified by univariate Cox regression analysis.

We have summarized the detailed flowchart in **Figure 1**. We downloaded and preprocessed the TCGA, GSE65858, and GSE41613 datasets to remove batch effects. Principal component analysis (PCA) was performed to determine whether the batch effects were removed. Before removing batch effects, samples from three different datasets formed distinct clusters (**Figure 2A**); however, after removing batch effects, they clustered together (**Figure 2B**). This suggests that batch effects can be effectively avoided by normalizing across the platforms. To avoid bias due to the short survival time, we excluded samples with no survival time or survival times less than 30 days and subsequently obtained clinical information and gene expression patterns of 854 individuals (TCGA: 491samples; GSE65858: 267 samples; GSE41613: 96 samples). After intersecting two ion channel gene sets (**Figure 2C**), 323 ion channel genes were obtained and are summarized in **Table S2**. Univariate Cox regression revealed that 20 genes were related to HNSCC prognosis (**Figure 2D**).

### Cluster analysis based on ion channel genes

**Figure 3:**
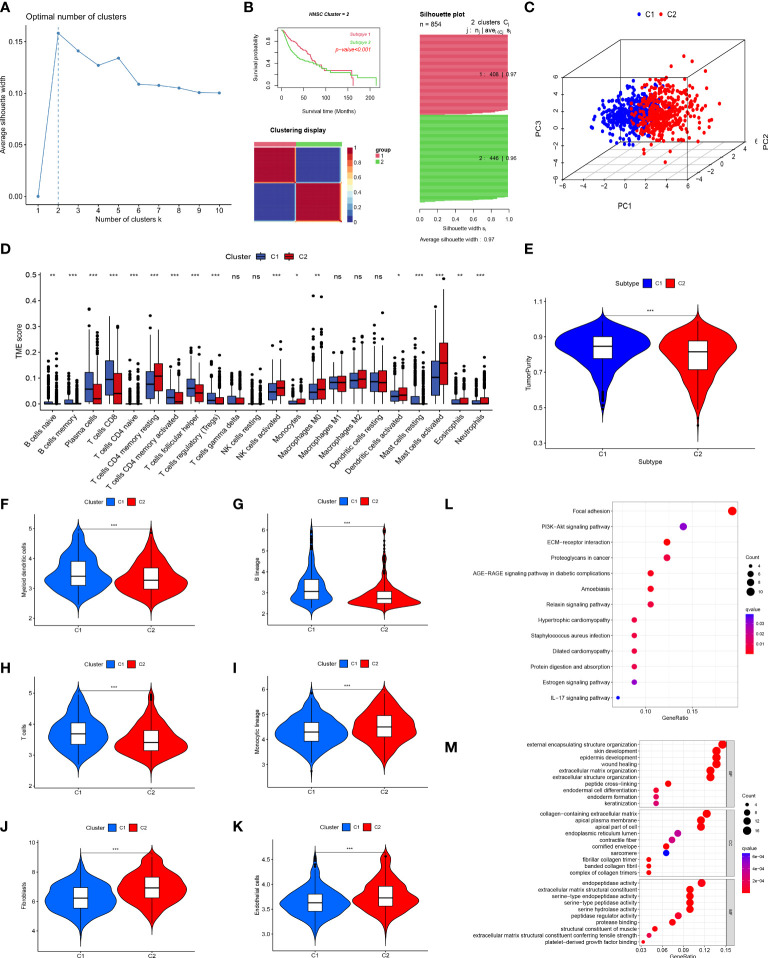
Tumor classification and immune infiltration analysis. **(A)** K-means determined that the optimal classification number was 2. **(B, C)** Consensus non-negative matrix factorization divided 854 patients into two clusters, with differences in OS. **(D)** The abundance of 23 infiltrating immune cell types in the two HNSCC subgroups. **(E)** The difference in tumor purity scores between the two subgroups. Comparison of myeloid dendritic cells **(F)**, B lineage cells **(G)**, T cells **(H)**, monocytic lineages **(I)**, fibroblasts **(J)**, and endothelial cells **(K)** between the two subgroups. **(L)** KEGG enrichment analysis of differential genes between the two subgroups. **(M)** GO enrichment analysis of differential genes between the two subgroups. (*p<0.05; **p<0.01; ***p<0.001; ns, no significant).

We used cluster analysis to stratify patients with HNSCC to investigate the overall prognostic significance of these genes. The results of the K-means method suggested that k=2 was the ideal number of subgroups (**Figure 3A**). Using the CNMF method, the 854 samples were separated into two subgroups, C1 (n=408) and C2 (n=446), and the K-M curve demonstrated an obvious distinction in OS among the two groups (P<0.001; **Figure 3B**), the **Figure 3C** shown the overall clustering of the two cancer subtypes.

The primary non-tumor components linked to disease outcomes in the tumor microenvironment (TME) are immune and stromal cells (33, 34). Using CIBERSORT, we evaluated the immune infiltration of these two clusters and discovered that many immune cells were differently distributed between these two subgroups, similar to the expression of CD8 T cells in subgroup C1. In contrast, activated mast cells were significantly expressed in subgroup C2 (**Figure 3D**). The ESTIMATE method was utilized to compare the tumor purity scores of the two subgroups, and it was found that the score of the C1 subgroup was higher, indicating that the C2 subgroup had a greater number of non-tumor cells (**Figure 3E**). In parallel, we used MCPcounter assays to compare the differences in immune cell infiltration between the two subgroups. The results of this analysis indicated that the distribution of immune cells differed between the two groups. Group C1 had more myeloid dendritic cells, B lineage, and T cells (**Figures 3F–H**), whereas group C2 had more monocytic lineages, fibroblasts, and endothelial cells (**Figures 3I–K**), which was supported by the results shown in **Figure 3D**.

We performed KEGG and GO enrichment analyses of the DEGs between the two clusters to further investigate various molecular pathways. KEGG pathway analysis showed that the DEGs were primarily involved in cancer-related focal adhesions, ECM-receptor interactions, and proteoglycans (**Figure 3L**). Furthermore, GO enrichment analysis demonstrated that these genes have active roles in external encapsulation, structural organization, collagen-containing extracellular matrix, apical plasma membrane, and endopeptidase activity (**Figure 3M**).

### Construction and validation of the 12-ion-channel-gene risk signatures

**Figure 4:**
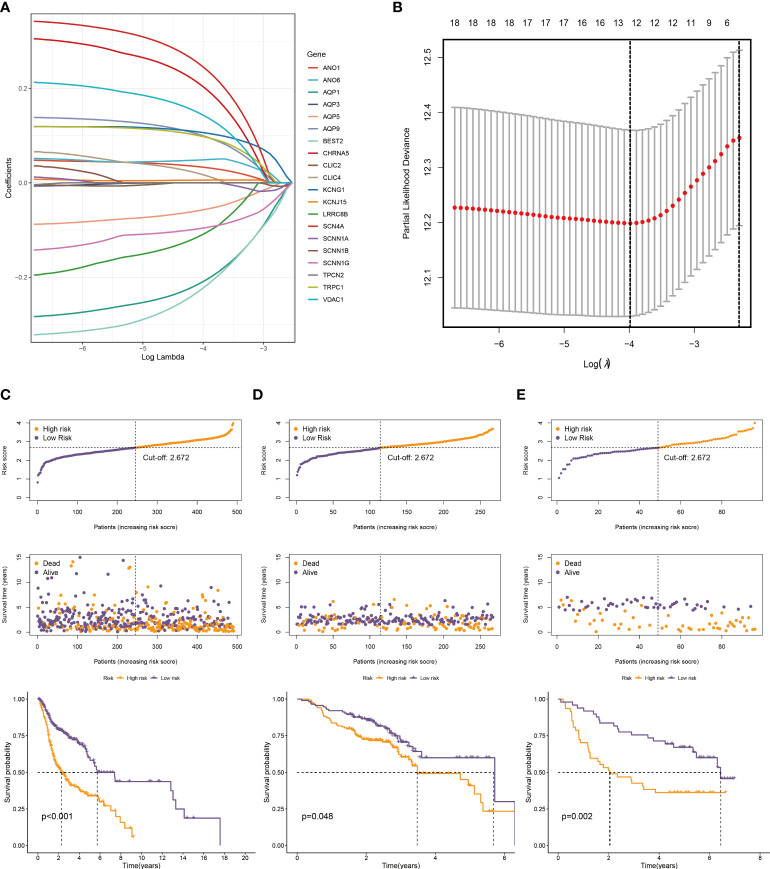
Construction of a prognostic model. **(A)** The LASSO coefficient profiles of the 20 ion channel genes. **(B)** Tuning parameter (λ) selection cross-validation error curve, the optimal log λ value is the left dotted line in the plot. The distribution of risk score, overall survival time, and OS status in the TCGA **(C)**, GSE65858 **(D)**, and GSE41613 cohorts **(E)**. The dotted line indicates the median point for risk score used to stratify patients into low-risk and high-risk groups, and the survival time of low-risk patients in the three datasets was longer.

Twenty genes were further analyzed using LASSO regression analysis, and 12 were screened to establish a prognostic signature (**Figures 4A, B**). The expression patterns of the 12 ion channel genes were multiplied by the associated LASSO coefficients to obtain risk scores. The detailed formula is as below: Score = (0.0418 * ANO1) + (-0.2274 * AQP1) + (-0.071 * AQP5) + (0.1130 * AQP9) + (-0.3912 * BEST2) + (0.1945 * CHRNA5) + (0.0751 * KCNJ15) + (0.1902 * KCNG1) + (-0.0261 * SCNN1G) + (0.2879 * SCN4A) + (0.1546 * TRPC1) + (0.2400 * VDAC1).

**Figure 5:**
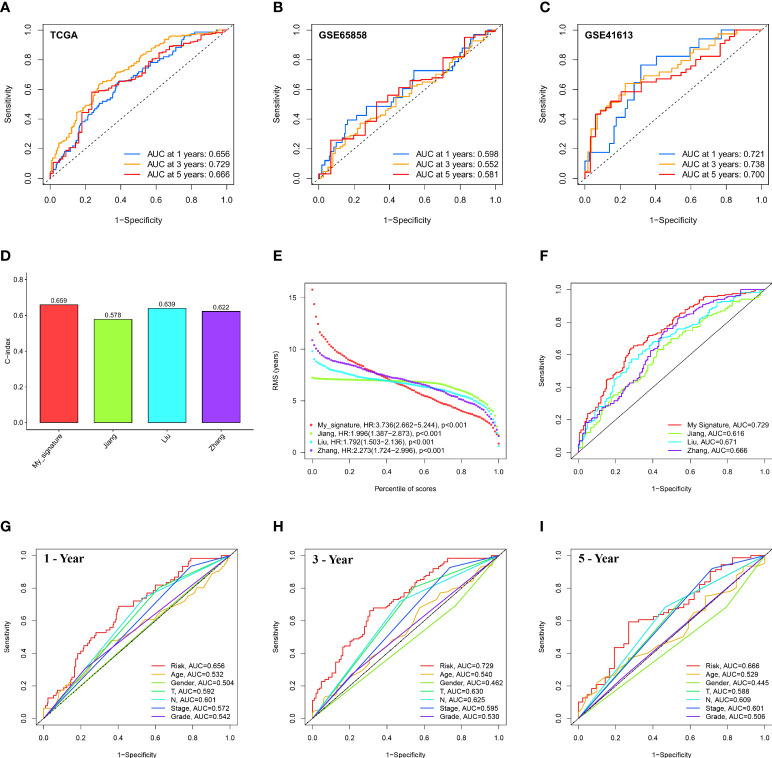
Evaluation and comparison of 12-ion-channel-gene model. ROC curve analysis of the 12-ion-channel-gene signature of the 1, 3, and 5 years in the TCGA **(A)**, GSE65858, and **(B)** GSE41613 datasets **(C)**. Comparison of our prognostic risk model with Jiang's, Liu's, and Zhang's in the TCGA cohort, **(D)** the C-indexes, **(E)** restricted mean survival time curves, **(F)** ROC curves. ROC curve analysis of risk score and the other six clinical features at 1 **(G)**, 3 **(H)**, and 5 years **(I)**.

We generated the corresponding risk scores for all samples in the TCGA dataset. Patients with HNSCC were divided into high- and low-risk groups based on the median risk score (**Figure 4C**). As risk scores increased, patients had a higher risk of death and shorter survival time. According to the K-M curve, the OS of high-risk patients was lower than that of low-risk patients (**Figure 4C**). Subsequently, we used the same approach to validate the GSE65858 and GSE41613 cohorts with the same results (**Figures 4D, E**). The time-dependent ROC curve was used to evaluate signature ability. At 1, 3, and 5 years, the AUCs of the TCGA group were 0.659, 0.729, and 0.666, respectively (**Figure 5A**). Comparatively, the 1-, 3-, and 5-years areas under the curve (AUCs) for GSE65858 were 0.598, 0.552, and 0.581, respectively (**Figure 5B**), whereas they were 0.721, 0.738, and 0.700 for GSE41613 (**Figure 5C**). The prediction effect of the GSE65858 cohort is not good enough, but lower AUC values in individual validation sets are acceptable because the distribution of disease characteristics between validation sets and training sets is different, such as the pathological stage of patients and regional differences. Our model shows a consistent trend in the survival analysis of different cohorts; that is, the high-risk group always has poor survival, so the signature can be considered reliable and repeatable.

In the TCGA cohort, we compared our 12-ion-channel-gene risk signature with Jiang's (27), Liu's (28), and Zhang's (29) models to judge their superiority. The C-index of all prognostic models was calculated using the “rms” package, and the results showed that our model had a higher C-index (0.659; **Figure 5D**). The predictive efficacy of the model at different time periods was also tested using restricted mean survival (RMS), and the results showed that our risk signature performed best over a 7-year period (**Figure 5E**). Our ROC curve had the highest AUC (0.729) among all the models (**Figure 5F**). Subsequently, **Figures 5G–I** shows that our signature has a higher prognostic value than traditional models. According to our findings, the 12-ion-channel-gene risk signature can somewhat predict the prognosis of HNSCC. Although the C-index is 0.659, its performance is still better than that of some existing gene models and worth paying attention to.

### Association between risk signature and clinicopathological features

**Figure 6:**
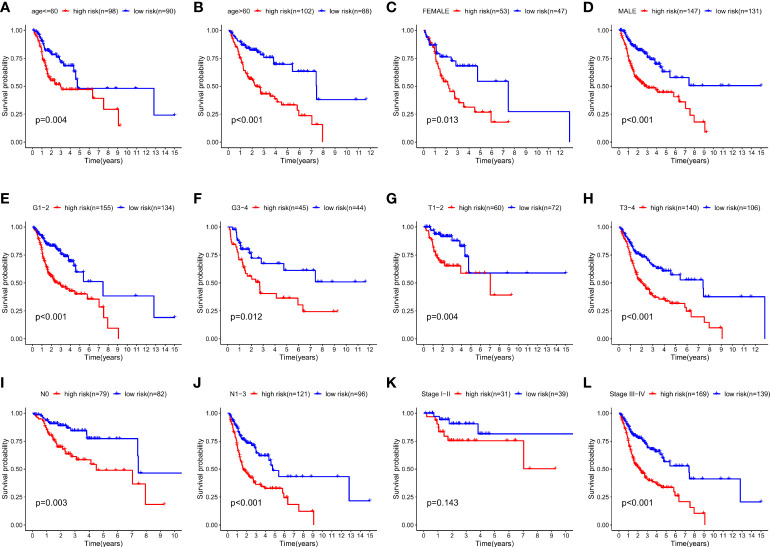
Kaplan-Meier survival curves for the high- and low-risk groups stratified by clinical factors. **(A)** Age ≤ 60 years, **(B)** Age > 60years, **(C)** gender (female), **(D)** gender (male), **(E)** T1-2, **(F)** T3-4, **(G)** N0, **(H)** N1-3, **(I)** stage I-II, **(J)** stage III-IV, **(K)** grade 1-2, and **(L)** grade 3-4.

**Figure 7:**
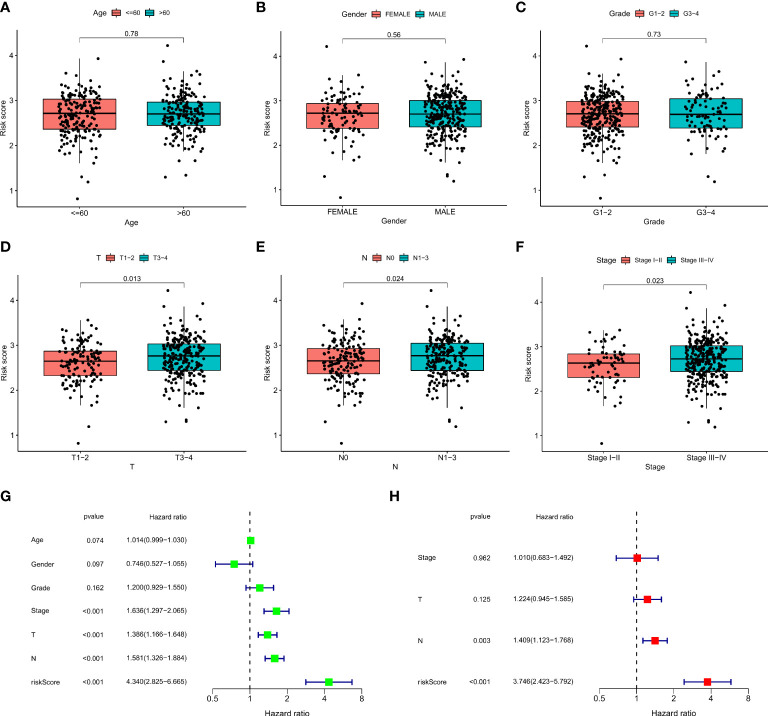
The correlations between the risk score and clinical factors **(A)** age, **(B)** gender, **(C)** T, **(D)** N, **(E)** stage, **(F)** and grade. Univariate Cox regression **(G)** and multivariate Cox regression analyses **(H)** of risk score and other clinical features were performed in the TCGA dataset.

We plotted the survival curves of HNSCC patients according to clinical risk variables (age, gender, grade, T, N, and stage; **Figures S1A–F**) and found obvious differences in OS among the T, N, and stage groups (**Figures S1D–F**). To evaluate the relationship between the 12-ion-channel-gene prognostic model and the above clinicopathological features, we first stratified the patients according to age (**Figures 6A, B**), gender (**Figures 6C, D**), grade (**Figures 6E, F**), T (**Figures 6G, H**), N (**Figures 6I, J**), and stage (**Figures 6K, L**). After stratification, the OS was significantly shorter in all high-risk groups, except for the stage (I-II) subgroup (**Figure 6K**). We then investigated the distribution of risk scores according to clinical characteristics. As shown in **Figures 7A–C**, the distribution of risk scores did not vary significantly by age, gender, or grade. However, the T3-4, N1-3, and stage III-IV risk scores were significantly higher than the T1-2, N0, and stage I-II risk scores (P<0.05; **Figures 7D–F**). Univariate and multivariate Cox analyses showed that the risk score was an independent prognostic factor of OS (univariate: hazard ratio [HR]: 4.340, 95% confidence interval [CI]: 2.826-6.665, P<0.001; **Figure 7G**; multivariate: HR: 3.746, 95% CI: 2.423–5.792, P<0.001; **Figure 7H**).

### Construction and evaluation of nomogram

**Figure 8:**
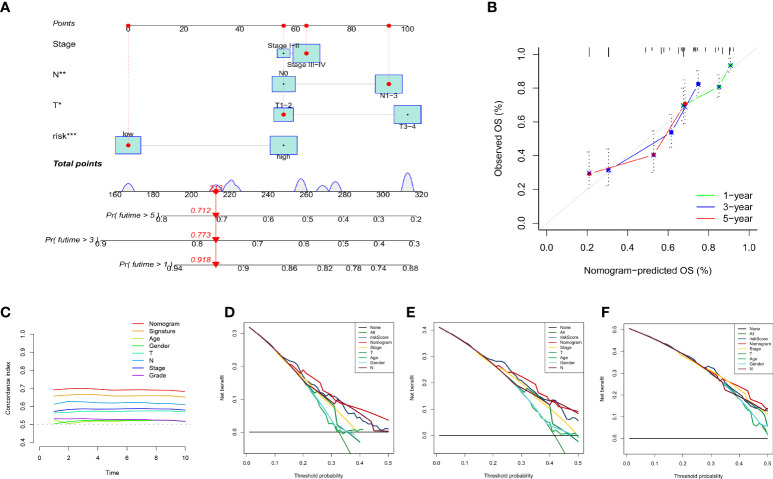
Establishment and evaluation of nomogram model in the TCGA dataset. **(A)** The nomogram for predicting the OS of patients at 1, 3, and 5 years. **(B)** Calibration curves of the nomogram for 1, 3, and 5 years. **(C)** The concordance index of the nomogram and other common clinical factors, the nomogram, and the signature showed superiority. Decision curve analysis at 1 **(D)**, 3 **(E)**, and 5 **(F)** years. The X-axis shows different thresholds.

We created a nomogram incorporating clinical indicators (stage, N, and T) and risk scores to better predict survival time in patients with HNSCC (**Figure 8A**). The calibration plot shows the difference between the nomogram predictions and the actual probability of survival in patients with HNSCC (**Figure 8B**), indicating the good prediction accuracy of the nomogram. As shown in **Figure 8C**, the C-index of the nomogram was much higher than that of each element, indicating strong predictive power. DCA of the nomogram revealed that the model had an outstanding net benefit for the 1, 3, and 5 years OS predictions (**Figures 8D–F**), which showed that the nomogram might be utilized as a useful tool to predict the outcome of patients in clinical practice.

### Mutation spectrum of 12 ion channel genes in HNSCC

**Figure 9:**
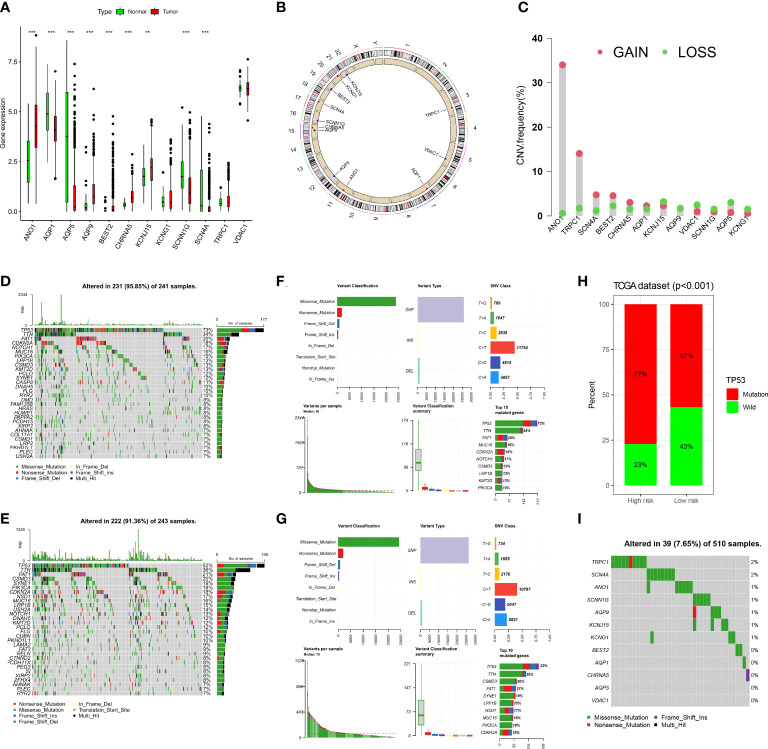
The landscape of genetic and expression variation of 12 ion channel genes in TCGA dataset. **(A)** Comparison of expression levels of 12 ion channel genes in tumor tissues and normal tissues. (**p<0.01, ***p<0.001). **(B)** The location of 12 ion channel genes on the chromosome. **(C)** Copy number variation of 12 genes. **(D, E)** Somatic mutations in the high-risk group. **(F, G)** Somatic mutation in the low-risk group. **(H)** Mutation difference of TP53 between the two groups. **(I)** The somatic mutation of 12 ion channel genes.

In the TCGA-HNSC cohort, we observed the expression of 12 ion channel genes in tumor and normal tissues; ANO1, AQP9, BEST2, CHRNA5, and KCNJ15 were upregulated in HNSCC, while AQP1, AQP5, SCNN1G, and SCN4A were downregulated (**Figure 9A**). We also discuss the frequency of CNVs and somatic mutations in 12 ion channel genes in HNSCC. The chromosomal locations of these genes are shown in **Figure 9B**, and the frequencies of CNV mutations are summarized in **Figure 9C**. In the high-risk group, 231/241 (95.85%) HNSCC samples exhibited gene mutations (**Figure 9D**), whereas in the low-risk group, 222/243 (91.36%) HNSCC samples exhibited gene mutations (**Figure 9E**). The most prevalent variant classification in the high-risk group was missense mutations, with SNPs being the most prevalent and C > T being the most frequent. The findings also showed that TP53 had the highest mutation frequency, followed by TTN (**Figure 9F**). The same conclusion was reached for the low-risk group (**Figure 9G**). More than half of the TP53 gene was mutated in the TCGA group, and the percentage of TP53 gene mutations was higher in the high-risk group (**Figure 9H**). Among the HNSCC samples in the TCGA dataset, the mutation frequency of the 12 ion channel genes was 7.65%, and TRPC1 and SCN4A had the highest mutation frequency (**Figure 9I**).

### Association between risk scores and tumor immunity

**Figure 10:**
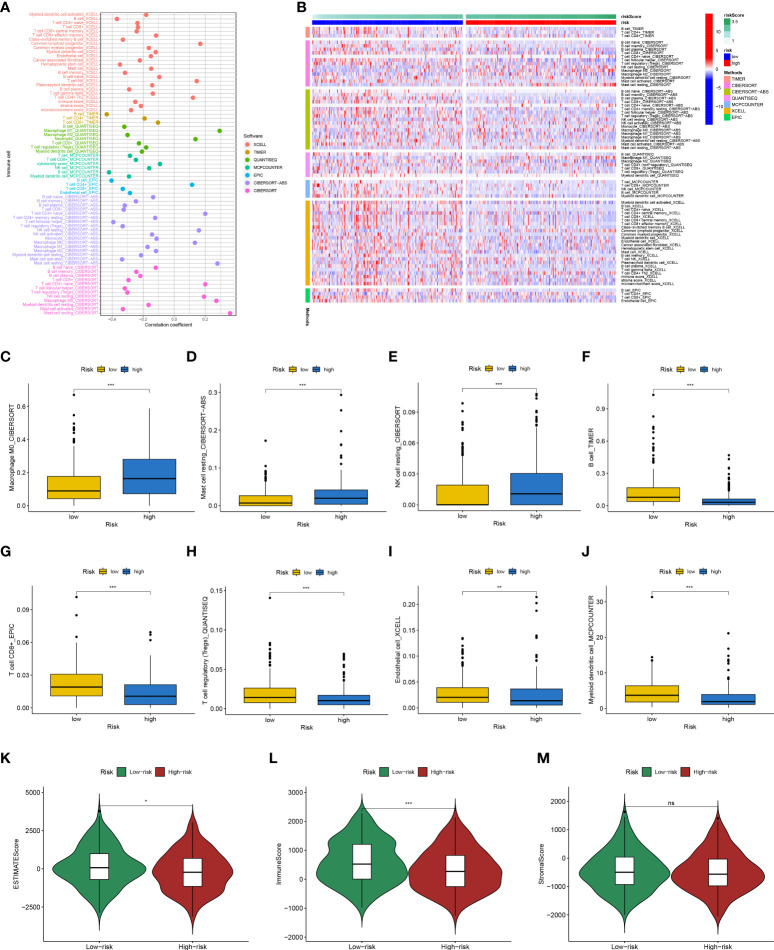
Analysis of immune landscape between the high-risk and low-risk groups in the TCGA dataset. **(A)** Overview of the association among risk scores and immune cells and stromal cells shown by Spearman correlation analysis. **(B)** Overview of immune cell differences between the two groups. The higher risk score was positively associated with the presence of tumor-infiltrating immune cells, including M0 macrophages **(C)**, resting mast cells **(D)**, and resting NK cells **(E)**, as well as was negatively associated with the presence of B cells **(F)**, CD8 + T cells **(G)**, regulatory T cells **(H)**, endothelial cells **(I)**, and myeloid dendritic cells **(J)**. Differences in estimate Score **(K)**, immune Score **(L)**, and stromal Score **(M)** from ESTIMATE algorithms between two groups. (*p<0.05; **p<0.01; ***p<0.001; ns, no significant).

Previous studies have shown that ion channels play a crucial role in immune cell infiltration. Therefore, we explored whether this risk signature is linked to TME. We analyzed immune cell infiltration using seven algorithms, and Spearman correlation was used to assess the correlation between risk scores and the TME (**Figure 10A**). A heat map depicting the relationship between immune infiltration and risk score is shown in **Figure 10B**. Among them, the risk score was positively correlated with the infiltration levels of M0 macrophages (**Figure 10C**), resting mast cells (**Figure 10D**), and resting NK cells (**Figure 10E**) and was negatively correlated with those of B cells (**Figure 10F**), CD8 + T cells (**Figure 10G**), regulatory T cells (**Figure 10H**), endothelial cells (**Figure 10I**), and myeloid dendritic cells (**Figure 10J**). The ESTIMATE results (**Figures 10K-M**) showed that the low-risk group had higher estimate and immune scores, indicating that the low-risk group had greater immune cell infiltration.

**Figure 11:**
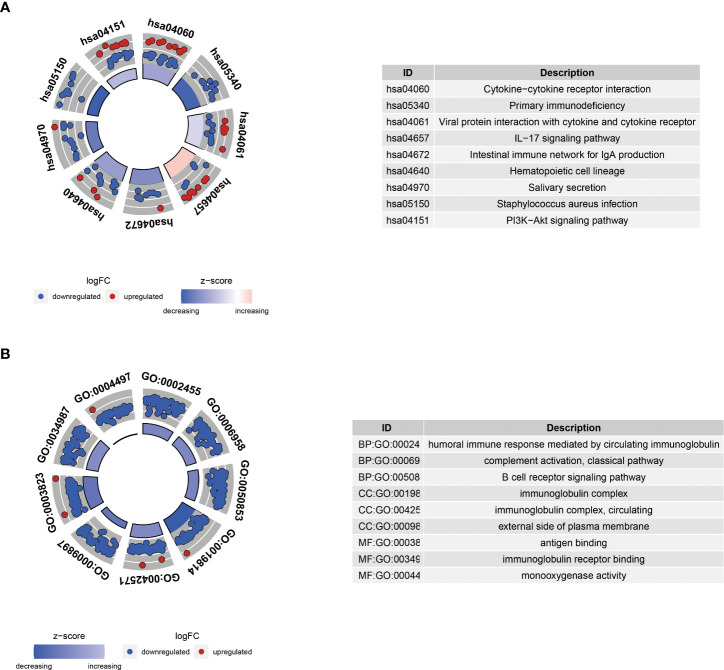
KEGG **(A)** and GO **(B)** enrichment analyses of differential genes between high-risk and low-risk groups. BP, biological process; CC, cellular component; MF, molecular function.

Besides, KEGG analysis showed that DEGs were primarily involved in cytokine-cytokine receptor interaction and primary immunodeficiency (**Figure 11A**). GO analysis demonstrated that these genes were mainly enriched in humoral immune response mediated by circulating immunoglobulins, immunoglobulin complexes, and antigen binding (**Figure 11B**).

### Risk scores predict immunotherapy response and chemosensitivity of HNSCC patients

**Figure 12:**
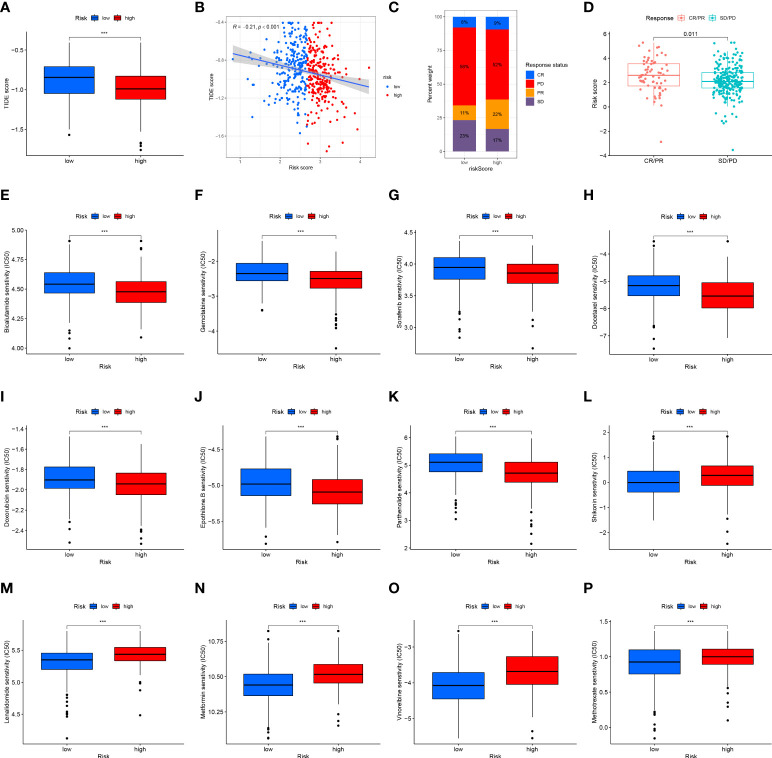
Risk score predicts responses to immunotherapy and chemotherapy. **(A)** Differences in the TIDE scores between the high-risk and low-risk groups in the TCGA dataset. **(B)** Scatter plots depicting the negative correlation between TIDE scores and risk scores in the TCGA dataset by the Spearman correlation analysis. **(C)** The proportion of patients in the IMvigor210 cohort with clinical response in high-risk and low-risk groups. CR, complete response; PD, progressive disease; PR, partial response; SD, stable disease. **(D)** Differences in the risk score among patients with different clinical responses in the IMvigor210 cohort. The box plot of the estimated IC50 for **(E)** bicalutamide, **(F)** gemcitabine, **(G)** sorafenib, **(H)** docetaxel, **(I)** doxorubicin, **(J)** epothilone B, **(K)** parthenolide, **(L)** shikonin, **(M)** lenalidomide, **(N)** metformin, **(O)** vinorelbine, and **(P)** methotrexate are shown between two groups in the TCGA dataset. The lower the IC50, the more sensitive the drug. (***p<0.001).

The TIDE algorithm was used to predict the likelihood of a response to immunotherapy. The results demonstrated that the high-risk group had significantly lower TIDE scores (P<0.001, **Figure 12A**), and the risk score was negatively correlated with the TIDE score (cor =-0.21, P<0.001; **Figure 12B**). In the IMvigor210 cohort, we calculated the risk score for each patient according to our formula. A higher proportion of patients in the high-risk group responded to immunotherapy (**Figure 12C**), and the risk score was significantly higher in the complete/partial response group than in the stable/progressive disease group (**Figure 12D**).

Vinorelbine, sorafenib, lenalidomide, metformin, and doxorubicin are often used in the clinical treatment of HNSCC. Lenalidomide can activate the ability of immune cells to stimulate the synthesis of immune cytokines, and metformin may alter the TME. The Wilcoxon test was performed to compare differences in chemotherapy sensitivity between the two groups, measured by IC50 values. IC50 values of bicalutamide (**Figure 12E**), gemcitabine (**Figure 12F**), sorafenib (**Figure 12G**), docetaxel (**Figure 12H**), doxorubicin (**Figure 12I**), and epothilone. B (**Figure 12J**) and parthenolide (**Figure 12K**) had higher IC50 values, while the IC50 value of shikonin (**Figure 12L**), lenalidomide (**Figure 12M**), metformin (**Figure 12N**), vinorelbine (**Figure 12O**), and methotrexate (**Figure 12P**) was lower. These results suggest that the 12-ion-channel-gene risk signature may be related to the response of HNSCC patients to immunotherapy and chemotherapy.

### Validation of target gene mRNA and protein in tumor and adjacent normal tissues

**Figure 13:**
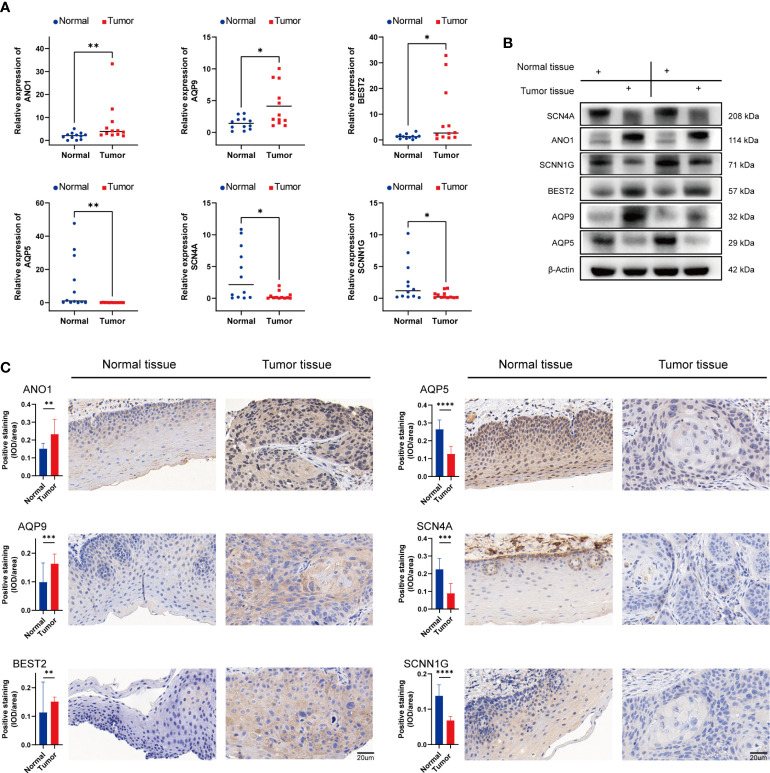
Validation of mRNA and protein expression levels of ion channel genes in HNSCC. **(A)** Comparison of mRNA expression levels of genes in 12 paired HNSCC tissues and adjacent normal tissue samples by qRT-PCR. **(B)** The western blot analyses. **(C)** The immunohistochemical staining in 12 paired HNSCC tissues and adjacent normal tissues. IOD, integrated optical density. (*p<0.05, **p<0.01, *** p<0.001, ****p<0.0001).

The nine genes with statistical significance in **Figure 9A** was verified by experiments. The mRNA expression of ANO1, AQP1, AQP5, AQP9, BEST2, SCNN1G, SCN4A, KCNJ15, and CHRNA5 in human head and neck cancer and normal adjacent tissues were confirmed by qRT-PCR. The findings revealed that ANO1, AQP9, and BEST2 were upregulated in tumor tissues, and AQP5, SCN4A, and SCNN1G expression was upregulated in normal tissue from 12 HNSCC patients (**Figure 13A**). The expression of CHRNA5, KCNJ15, and AQP1were not statistically significant (**Figure S2**), but the trend was the same as that of bioinformatics analysis. Then, ANO1, AQP9, BEST2, AQP5, SCN4A, and SCNN1G protein expression levels were confirmed by western blotting (**Figure 13B**) and immunohistochemistry staining (**Figure 13C**) and were compatible with mRNA expression levels.

## Discussion

HNSCCs are solid malignant tumors with high immunogenicity, and their global incidence is increasing annually. The local control rate and quality of life of HNSCC patients have improved owing to advances in surgical treatment methods and comprehensive treatment strategies, but the 5-year survival rate is only 40-50% (35). The standard of care for recurrent or metastatic HNSCC is platinum-based chemotherapy combined with cetuximab. However, there are problems such as simple recurrence and short median survival after treatment (36–38). PD-1/PDL-1 monoclonal antibody-based ICI therapy has emerged as a new treatment option for advanced HNSCC. Several tumors, including lung carcinoma (39), prostate carcinoma (40), colon carcinoma (41), and glioblastoma (42), have recently been found to express ion channel genes to varying degrees. It has been demonstrated that increased ion channel expression promotes malignant tumor progression. Therefore, the use of ion channels as indicators of cancer diagnosis and prognosis has received increasing attention. In this study, we used ion channel genes to construct an HNSCC risk signature and identify individuals with poor prognoses, which may aid in the clinical management of patients with HNSCC.

The expression and prognostic significance of ion channel genes in HNSCC were also investigated. In the TCGA dataset, when comparing HNSCC to normal tissues, we found that the expression of ANO1, AQP9, BEST2, CHRNA5, and KCNJ15 was higher in HNSCC, whereas the expression of AQP1, AQP5, SCNN1G, and SCN4A was lower, which was consistent with our experimental results.

The small sample size of the dataset can lead to poor accuracy. To this end, we included three datasets (TCGA, GSE65858, and GSE41613) with 854 tumor samples. We successfully divided the tumors into two subgroups using the CNMF method. According to the PCA results, our classification was robust. Tumor tissues generally include tumor cells, invading and resident host cells, the extracellular matrix, and secreted substances (43). The sustained growth, invasion, and metastasis of tumor cells depend on the TME, which plays a crucial role in tumor biology (44). The tumor immune score is a key determinant of tumor growth and immunotherapy success (45). In this study, we calculated the purities of two subgroups of tumors using the ESTIMATE method and observed immune infiltration. We observed changes in immune cell levels between the two clusters, which was surprising. We performed a functional enrichment analysis of the two subgroups to further explore their underlying differential processes. According to these findings, the differences between the two subgroups were mainly focused on the regulation of focal adhesions, ECM-receptor interaction, and proteoglycans. These functions and pathways participate in the progression of HNSCC. For example, a previous study found that ECM remodeling promotes cancer development and indicates poor prognoses in HNSCC patients (46). These findings imply that the role of ion channel genes in the occurrence and development of HNSCC requires further investigation.

In our study, the above 12 ion channel genes were found to be related to tumor prognosis, which is consistent with the results of previous studies. ANO1, also called TMEM16A, ORAOV2, DOG1, TAOS2, and FLJ10261, is a calcium-activated chloride channel, and its overexpression can promote the attachment, spread, detachment, and invasion of HNSCC cells and can be used as a marker for distant metastasis of HNSCC (47). The aquaporins (AQPs) are a family of small integral membrane proteins that facilitate water transport across the plasma membranes of cells in response to osmotic gradients (48). AQP1 is mainly expressed at the apical membrane of blood vessel endothelial cells and is upregulated in different carcinomas, including breast, lung, and colon carcinomas. Several reports have shown that AQP1 overexpression in tumor cells may increase tumor invasiveness and angiogenesis, all of which contribute to tumor expansion (49). AQP5 is expressed in the apical membrane of alveolar type I cells, and its overexpression leads to enhanced activation of the epidermal growth factor receptor, extracellular receptor kinase, and p38 mitogen-activated protein kinase pathways, which promote the proliferation and migration of lung cancer cells (50). Similar to AQP1, AQP9 is upregulated in clear cell renal cell carcinoma (ccRCC) tissues compared to normal tissues, and upregulation of AQP9 can correlate with aggressive progression and poor survival in ccRCC patients (51). CHRNA5 encodes the α5 nicotinic acetylcholine receptor subunit and is involved in the nicotine-induced proliferation of lung cancer cells (52). KCNJ15, a member of the J subfamily of potassium inward rectifier channels, is mainly expressed in the human kidney and pancreas and is a risk gene for type 2 diabetes; its downregulation promotes insulin secretion (53). Importantly, the overexpression of KCNJ15 was shown to significantly inhibit the proliferation, migration, and colony formation of renal cancer cells, arrest the cell cycle and induce cancer cell apoptosis, which may be a tumor suppressor gene in renal cancer (54). SCNN1G encodes a non-voltage-gated epithelial sodium channel that plays a key role in maintaining sodium and water homeostasis. In addition, SCNN1G is downregulated in cervical cancer and is negatively correlated with the histological grade of the tumor, and its overexpression is associated with better OS (55). SCN4A is the fourth member of the SCN family that encodes sodium channels, is highly expressed in hepatocellular carcinoma, and has varying degrees of correlation with tumor grade, lymph node metastasis status, and cancer stage (56). The TRPC subfamily constitutes a group of nonselective cation channels that mediate the influx of Ca2+ and other cations into the cytosol of cells and has been shown to emerge as a potential regulator of various physiological and pathological processes. As one of the seven mammalian members of the TPRCs, TRPC1 plays a pivotal role in colorectal tumorigenesis and tumor progression by activating the calmodulin-mediated PI3K/AKT signaling axis and may be a novel target for colorectal cancer treatment (57). VDAC1 is a voltage-dependent anion channel located in the outer mitochondrial membrane that mediates the transport of metabolites and ions into and out of mitochondria, participating in mitochondria-mediated apoptosis. It is highly expressed in chronic lymphocytic leukemia (CLL), and the VDAC1-based peptides are considered a potential and effective anti-CLL therapy (58). To the best of our knowledge, there are few published studies on the relationship between BEST2 and KCNG1 and tumors and their interaction with HNSCC.

Through integrated analysis of high-throughput data, we found a link between the 12-ion-channel-gene signature and immune infiltration. Our study showed that the levels of B cells, CD8 + T cells, regulatory T cells, myeloid dendritic cells, and endothelial cells in the low-risk group were higher, and the high-risk group had higher M0 macrophages, resting mast cells, and resting NK cells. A substantial body of research has revealed that dense infiltration of B cells, CD8 T cells, and dendritic cells indicates a favorable prognosis for tumors (59–61). M0 macrophages, also known as resting-state macrophages derived from bone marrow, are usually considered precursors of polarized macrophages. It was once thought that M0 was only a resting state of macrophages without a specific function before their polarization. However, recent studies have found that M0 macrophages are associated with poor prognosis in some tumors, such as lung adenocarcinoma (62). Similar to M0 macrophages, high densities of resting mast cells have also been found to be associated with poor prognosis in pancreatic cancer (63). The results of our study are consistent with these conclusions.

KEGG and GO functional enrichment analysis between the high- and low-risk groups showed that the DEGs mainly enriched in immune-related pathways, which may be related to the immunotherapy response of HNSCC. Meanwhile, the results of immunotherapy response prediction also confirmed this. The TIDE scores of the high-risk subtypes were lower, suggesting that patients in the high-risk group may respond better to immunotherapy, and the predicted results of the IMvigor210 cohort were also consistent. Therefore, we believe that our predictive model will provide useful tumor immunological assessments and treatment recommendations.

We observed many genetic mutations in both the high- and low-risk groups, including the most common TP53 gene mutation. The percentage of TP53 gene mutations in both groups was more than 50%, especially in the high-risk group, and the mutation rate of the TP53 gene was as high as 77%. The TP53 gene has been shown to have the highest number of mutations in HNSCC, and previous studies have demonstrated that TP53 affects the expression of ion channel genes (64). Furthermore, since TP53 mutation is the most prevalent single genetic event in cancer, it is related to poor outcomes in multiple tumors, which is consistent with our results.

Another important conclusion of our study was the relationship between risk scores and sensitivity to chemotherapeutics such as vinorelbine, sorafenib, lenalidomide, metformin, and doxorubicin. These findings showed considerable differences in drug sensitivity between the high- and low-risk groups. Therefore, the 12-ion-channel-gene signature may be helpful for immunotherapy and chemotherapy response prediction in HNSCC patients.

This study had some limitations. First, the study relied on public databases, although some experimental validation was performed, more in vivo and in vitro studies are required to corroborate our results. Second, this study was limited to ion channel genes and lacked an explanation of key pathways. However, these limitations did not affect the usefulness of this study.

In conclusion, we performed a comprehensive bioinformatics study of HNSCC and constructed 12 ion channel gene signatures (ANO1, AQP1, AQP5, AQP9, BEST2, CHRNA5, KCNJ15, KCNG1, SCNN1G, SCN4A, TRPC1, and VDAC1). This signature can indicate the mutational status, immune cell infiltration pattern, immunotherapy sensitivity, and chemosensitivity of HNSCC and may be used to predict prognoses and guide treatment.

## Data availability statement

The datasets generated during and/or analysed during the current study are available in The Cancer Genome Atlas (TCGA) database (https://portal.gdc.cancer.gov/), Gene Expression Omnibus (GEO-GSE65858 and GSE41613) database (http://www.ncbi.nlm.nih.gov/geo) and Genomics of Drug Sensitivity in Cancer (GDSC) (https://www.cancerrxgene.org/). The original contributions presented in the study are included in the article/Supplementary Material. Further inquiries can be directed to the corresponding author.

## Ethics statement

This study was approved by the Ethics Committee of the First Affiliated Hospital of the Anhui Medical University (number: Quick-PJ 2022-03-19). The patients/participants provided their written informed consent to participate in this study.

## Author contributions

All authors contributed to the study conception and design. HL and YH contributed to the conception of the study. BC, YS, JWi, YL, SS and ZF performed the data analyses and experimental verification. YH, YL, CS, XW and SY contributed significantly in writing the manuscript. All authors contributed to the article and approved the submitted version.

## Funding

This study was supported by Scientific Research Cooperation Project Between Anhui Medical University and Hefei First People's Hospital (K202003, chaired by Haiwen Li).

## Conflict of interest

The authors declare that the research was conducted in the absence of any commercial or financial relationships that could be construed as a potential conflict of interest.

## Publisher’s note

All claims expressed in this article are solely those of the authors and do not necessarily represent those of their affiliated organizations, or those of the publisher, the editors and the reviewers. Any product that may be evaluated in this article, or claim that may be made by its manufacturer, is not guaranteed or endorsed by the publisher.

## Supplementary material

Supplementary Figure 1 The Kaplan-Meier curves for the clinical factors including **(A)** age, **(B)** gender, **(C)** grade, **(D)** T, **(E)** N, and **(F)** stage.

Supplementary Figure 2 qRT-PCR analysis of CHRNA5 **(A)**, KCNJ15 **(B)**, and AQP1 **(C)** mRNA levels in tumor and normal tissues. ns = no significant.

Supplementary Table 2 The 323 ion channel genes.
