# Supplementary figures and images for: An ion-channel-gene-based prediction model for head and neck squamous cell carcinoma: Prognostic assessment and treatment guidance
Source: Front Immunol. 2022 Oct 28;13:961695. doi: 10.3389/fimmu.2022.961695 (PMC9650652; doi:10.3389/fimmu.2022.961695)

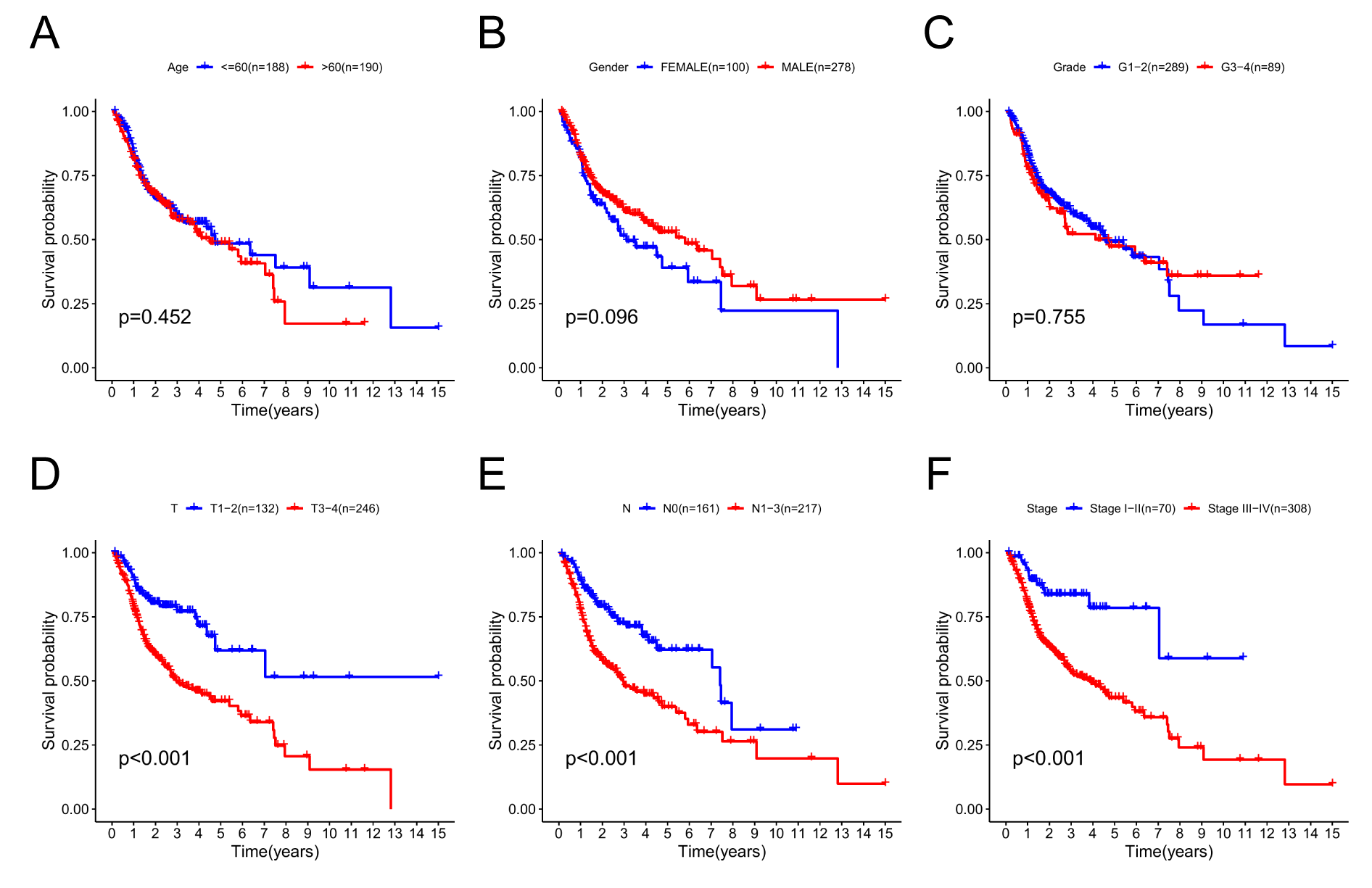

Supplement: Supplementary Figure 1 — The Kaplan-Meier curves for the clinical factors including (A) age, (B) gender, (C) grade, (D) T, (E) N, and (F) stage. [file Image_1.tif]

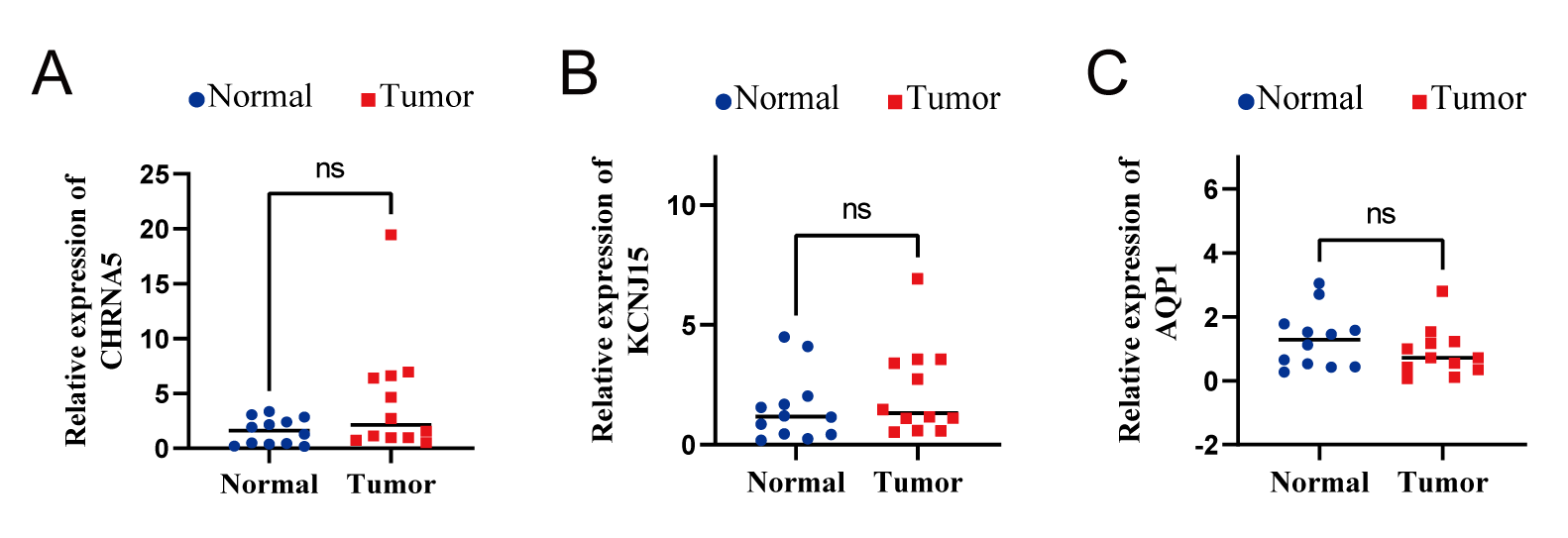

Supplement: Supplementary Figure 2 — qRT-PCR analysis of CHRNA5 (A), KCNJ15 (B), and AQP1 (C) mRNA levels in tumor and normal tissues. ns = no significant. [file Image_2.tif]
