# Supplementary material for: An ion-channel-gene-based prediction model for head and neck squamous cell carcinoma: Prognostic assessment and treatment guidance
Source: Front Immunol. 2022 Oct 28;13:961695. doi: 10.3389/fimmu.2022.961695 (PMC9650652; doi:10.3389/fimmu.2022.961695)
Supplement: Supplementary file 4 [file Table_1.docx]

**Supplemental Information**

**An ion-channel-gene-based prediction model for head and neck squamous cell carcinoma: prognostic assessment and treatment guidance**

**Yanxun Han, Yangyang Shi,** **Bangjie Chen,** **Jianpeng Wang, Yuchen Liu, Shuyan Sheng,** **Ziyue Fu,** **Chuanlu Shen,** **Xinyi Wang,** **Siyue Yin, Haiwen Li**

**Table S1.** **The primer sequences of qRT-PCR.**

| Primer | Sequence (5’-3’) |
| --- | --- |
| ANO1 F | CTGATGCCGAGTGCAAGTATG |
| ANO1 R | AGGGCCTCTTGTGATGGTACA |
| AQP9 F | GAAGAGCAGCTTAGCGAAAGA |
| AQP9 R | ACAGCCACATCCAAGGACAAT |
| BEST2 F | CGTGGGAGCATCTACAAACTC |
| BEST2 R | GTCAGCACAAAGCGGTAGG |
| KCNJ15 F | CCGCGTCATGTCCAAGAGT |
| KCNJ15 R | TGCAGGTAGAGTAGGTATATGCC |
| AQP1 F | CTGGGCATCGAGATCATCGG |
| AQP1 R | ATCCCACAGCCAGTGTAGTCA |
| AQP5 F | CGGGCTTTCTTCTACGTGG |
| AQP5 R | GCTGGAAGGTCAGAATCAGCTC |
| SCNN1G F | GCACCCGGAGAGAAGATCAAA |
| SCNN1G R | TACCACCGCATCAGCTCTTTA |
| CHRNA5 F | AAAGATGGGTTCGTCCTGTGG |
| CHRNA5 R | CAAACAAAACGATGTCTGGTGTC |
| SCN4A F | AGTCACTGGCAGCCATAGAAC |
| SCN4A R | CCTTGTTGAGTACGATGAAGGTC |
| GAPDH F | GGAGCGAGATCCCTCCAAAAT |
| GAPDH R | GGCTGTTGTCATACTTCTCATGG |

**Table S2.** **The 323 ion channel genes (submitted as a separate Excel file).**
